# Methamphetamine potentiates HIV-1 gp120-mediated autophagy via Beclin-1 and Atg5/7 as a pro-survival response in astrocytes

**DOI:** 10.1038/cddis.2016.317

**Published:** 2016-10-20

**Authors:** Lu Cao, Mingui Fu, Santosh Kumar, Anil Kumar

**Affiliations:** 1 Division of Pharmacology and Toxicology, School of Pharmacy, University of Missouri-Kansas City, Kansas City, MO, USA; 2 Department of Basic Medical Science, School of Medicine, University of Missouri-Kansas City, Kansas City, MO, USA; 3 Department of Pharmaceutical Sciences, College of Pharmacy, University of Tennessee Health Science Center, Memphis, TN, USA

## Abstract

Methamphetamine (METH), a commonly used controlled substance, is known to exacerbate neuropathological dysfunction in HIV-infected individuals. The neuropathological manifestation results from cell death or dysfunction in the central nervous system (CNS) wherein autophagy is expected to have an important role. Autophagy is generally considered protective during deprivation/stress. However, excessive autophagy can be destructive, leading to autophagic cell death. This study was designed to investigate if METH and HIV-1 gp120 interact to induce autophagy in SVGA astrocytes, and whether autophagy is epiphenomenal or it has a role in METH- and gp120-induced cytotoxicity. We found that METH and gp120 IIIb caused an increase in LC3II level in astrocytes in a dose- and time-dependent manner, and the level of LC3II was further increased when the cells were treated with METH and gp120 IIIb in combination. Next, we sought to explore the mechanism by which METH and gp120 induce the autophagic response. We found that METH induces autophagy via opioid and metabotropic glutamate receptor type 5 (mGluR5) receptors. Other than that, signaling proteins Akt, mammalian target of rapamycin (mTOR), Beclin-1, Atg5 and Atg7 were involved in METH and gp120-mediated autophagy. In addition, long-term treatment of METH and gp120 IIIb resulted in cell death, which was exacerbated by inhibition of autophagy. This suggests that autophagy functions as a protective response against apoptosis caused by METH and gp120. This study is novel and clinically relevant because METH abuse among HIV-infected populations is highly prevalent and is known to cause exacerbated neuroAIDS.

Although the introduction of highly active antiretroviral therapy (ART) has significantly reduced the incidence of HIV-associated dementia (HAD), HIV-associated neurocognitive disorders (HAND) remains a major problem in sizeable number of infected individuals.^1, 2^ This is compounded by the fact that mechanism of HIV neuropathogenesis is poorly understood. There are three major cell types in the brain of which only microglia and astrocytes are susceptible to viral infection. Astrocytes comprise approximately 70% of the brain, and are thought to have an important role in pathogenesis of HAND.^3^ The interactions between astrocytes and neurons are crucial for neuronal survival under the pathological condition. An impairment in the functions of astrocytes can negatively impact the neurons, leading to neurodegenerative diseases. For example, apoptotic astrocytes has been found in the brains of patients with HAD.^4^ It is generally considered that HIV-1 induces neurotoxicity via direct as well as indirect effects of the viral proteins on astrocytes.^5^ We among others have earlier shown that variety of HIV proteins including HIV-1 gp120,^6, 7, 8, 9^ Tat,^10, 11^ Nef^12, 13^ and Vpr^14^ can cause central nervous system (CNS) toxicity by exerting their effect on astrocytes.

Methamphetamine (METH) is one of the most commonly used recreational drugs in the United States, and its prevalence is increased in HIV-infected population (~15%) compared with the normal population.^15^ It is a potent psychostimulant that causes neurotoxicity via several mechanisms such as damage of both dopamine and serotonin neurons in the CNS,^16, 17^ induction of oxidative stress^18^ and dysregulation of glutamate uptake in CNS.^19^ In addition, the use of METH also leads to clinical symptoms such as rapid and irregular heartbeat, delirium, psychosis and heart failure.^20, 21^ METH has been shown to exacerbate HIV-associated neurotoxicity in the CNS.^22^ Previous studies have proved that METH and gp120 work synergistically to increase the level of proinflammatory cytokine IL-6 and induce oxidative stress, which lead to apoptosis in astrocytes.^23, 24^

Autophagy is a regulated degradative process in eukaryotic cells that allows recycling of cellular components under stress condition, and protects the cells from dying.^25^ During this process, cytoplasmic organelles are sequestered within autophagosome and delivered into lysosomes to be degraded by acidic lysosomal hydrolases. Autophagy also functions as a housekeeping process that removes misfolded proteins and damaged organelles. In addition, autophagy pathway can sometimes be utilized by viruses to promote their own replication.^26^ Autophagy is initiated when stress signal is received from mammalian target of rapamycin (mTOR). mTOR activity is inhibited under stress condition, leading to activation of downstream pathway of autophagy-related proteins such as Atg1, Beclin-1, Atg5 and Atg7. The formation of autophagosome requires microtubule-associated protein-1 light chain 3 (MAPLC3), which is commonly used as the marker of autophagy.

Autophagy is generally considered to be a survival mechanism, which preserves the balance between protein synthesis, organelle biogenesis and their clearance. Autophagy has also been linked to non-apoptotic cell death, called type II programmed cell death, or autophagic cell death.^27^ Extensive autophagy has been reported when cells are exposed to various drugs of abuse.^28, 29, 30, 31^ For example, our recent work has demonstrated that autophagy is associated with cocaine-induced astrocytic cell death.^29^ Similarly, autophagy has been detected when cells are exposed to low doses of METH, and suppression of the autophagy is associated with cell death.^28, 30^ Autophagy has also been found to be involved in several neurodegenerative diseases such as Alzheimer's disease, Parkinson's disease and Huntington's disease.^32, 33, 34^ However, the role of autophagy in HIV-induced neurotoxicity is poorly understood. This study was designed to determine the combined effects of METH and gp120 on autophagy in astrocytes, and the underlying mechanism of the METH- and gp120-mediated autophagy.

## Results

### METH and gp120 IIIb induce autophagy in astrocytes

**Figure 1:**
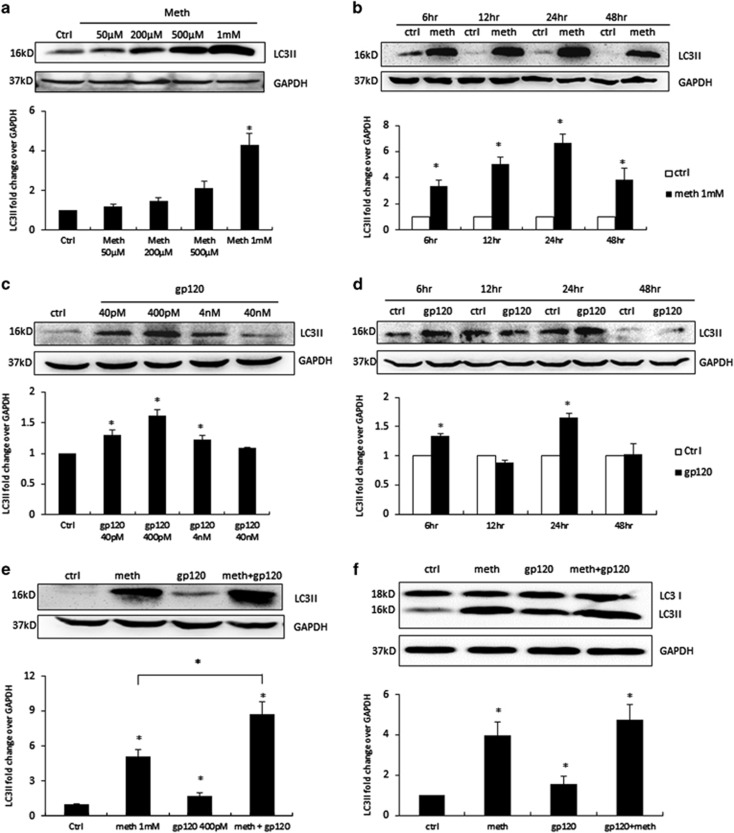

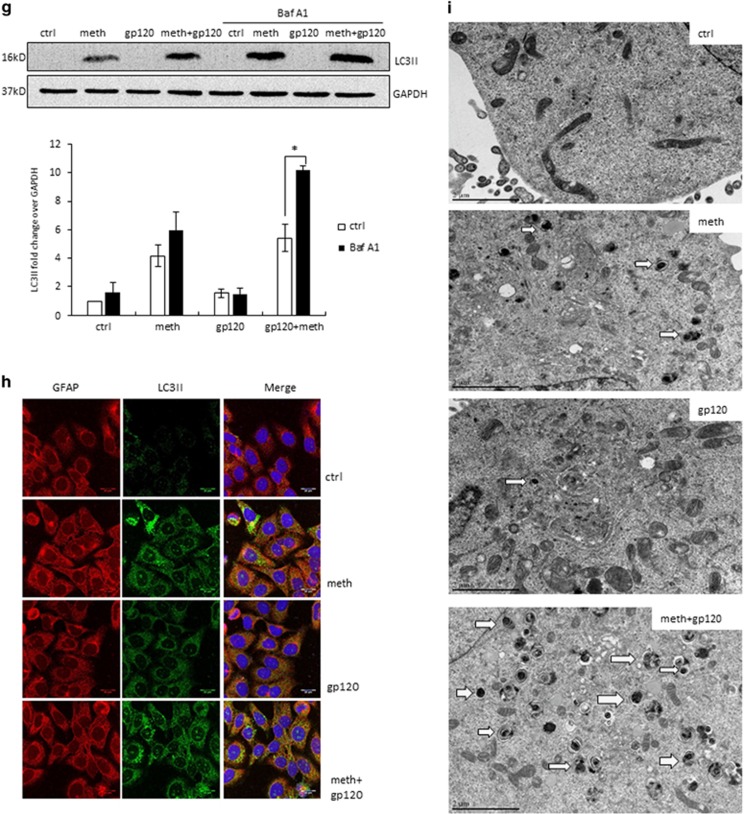
METH and HIV-1 gp120 IIIb induce autophagy in astrocytes. LC3II was analyzed using the western blot and quantified by AlphaEase FC software (Alpha Innotech, San Leandro, CA, USA), which are shown at the bottom of each panel (**a**–**f**). Results are shown as mean±S.E. from three separate experiments. *P<0.05. (**a**) SVGA cells were exposed to different doses of METH for 24 h, (**b**) SVGA cells were exposed to 1 mM of METH at varying time periods, (**c**) SVGA cells were exposed to different doses of HIV-1 gp120 IIIb for 24 h, (**d**) SVGA cells were exposed to 400 pM gp120 IIIb at varying time periods, (**e**) SVGA cells were exposed to 1 mM of METH, 400 pM of gp120 IIIb, or both for 24 h, (**f**) human primary astrocytes were treated with METH and gp120 IIIb as indicated, (**g**) SVGA cells were exposed to bafilomycin A1 1 h before treatment of METH, gp120 IIIb, or both for 24 h. (**h**) LC3II punctate dots in METH- and gp120- treated SVGA cells. SVGA cells were treated with 1 mM of METH, 400 pM of gp120 IIIb, or both for 24 h. Cells were fixed with ice-cold methanol: acetone (1 : 1), immunostained with anti-LC3II antibody, and examined by confocal microscopy (scale bar, 20 *μ*m). (**i**) Electron microscopy images showing the ultrastructure of METH- and gp120-treated SVGA cells. Arrows in the electron micrograph denote presence of autophagosomes (scale bar, 2 *μ*m). Immunostaining and microscopic images are representatives of at least three independent experiments

During autophagy, the cytosolic form of LC3 (LC3I) is conjugated to phosphotidylethanolamine to form LC3I-phosphotidylethanolamine complex (LC3II), which is widely accepted as the marker for autophagy activity. To determine whether METH and gp120 IIIb can induce autophagy in astrocytes, we treated SVGA astrocytes with varying concentrations of METH for 24 h, followed by the western blot analysis of LC3II in whole-cell lysate. SVGA cells responded to METH treatment in a dose-dependent manner, with 1 mM of METH showing maximum increase in LC3II level (~4-fold) (Figure 1a). SVGA cells were then treated with 1 mM of METH for varying time periods. METH caused significant increase in LC3II level starting from 6 h, and peaked at 24 h (~6-fold) (Figure 1b). Similarly, to determine whether gp120 IIIb will induce autophagy in astrocyte, we treated cells with different concentrations of gp120 IIIb as indicated for 24 h. The gp120 IIIb caused an increase in LC3II level in SVGA cells, with 400 pM of gp120 IIIb showing maximum increase in LC3II (~1.5-fold) (Figure 1c). Following that, to determine the optimal time point for gp120 IIIb-induced autophagy, we treated cells with 400 pM of gp120 IIIb at varying time periods. Interestingly, gp120 IIIb caused initial increase in LC3II level at 6 h, and a subsequent decrease at 12 h followed by a peak at 24 h (1.6-fold) post-exposure (Figure 1d).

As METH and gp120 IIIb individually induce autophagy in SVGA cells, we next examined whether they will show combination effect in inducing autophagy. We treated SVGA cells with 1 mM of METH and 400 pM of gp120 IIIb individually and in combination. Surprisingly, METH and gp120 IIIb together showed further increase in the level of LC3II (~8-fold)compared with METH (~4.5-fold) or gp120 IIIb (1.5-fold) alone (Figure 1e). To confirm this result, same treatment was applied to human primary astrocytes, which also yielded similar results (Figure 1f). As increased level of LC3II can be observed when autophagy is either induced or inhibited, a fusion inhibitor, bafilomycin A1, was used to exclude the possibility of defect in lysosomal degradation. Pretreatment of bafilomycin A1 caused further increase in the level of LC3II following METH and combination treatment (Figure 1g). The increase in autophagy level was further confirmed by immunocytochemistry in SVGA cells upon the treatment of METH and gp120 IIIb (Figure 1h). There was a substantial increase in LC3II-positive puncta when the cells were treated with METH and gp120 IIIb in combination compared with individual treatment. Furthermore, we examined the formation of autophagosomes by transmission electron microscopy in the SVGA (Figure 1i). The results showed numerous multi-membrane vacuoles (autophagosomes) in the combination treatment, whereas few or no autophagosomes were observed in the control, METH-, or gp120-treated cells. Taken together, these results clearly showed that METH and gp120 induce autophagy in astrocytes in additive manner.

### Signaling proteins mTOR, Beclin-1, Atg5 and Atg7 are involved in METH- and gp120-induced autophagy in astrocytes

**Figure 2:**
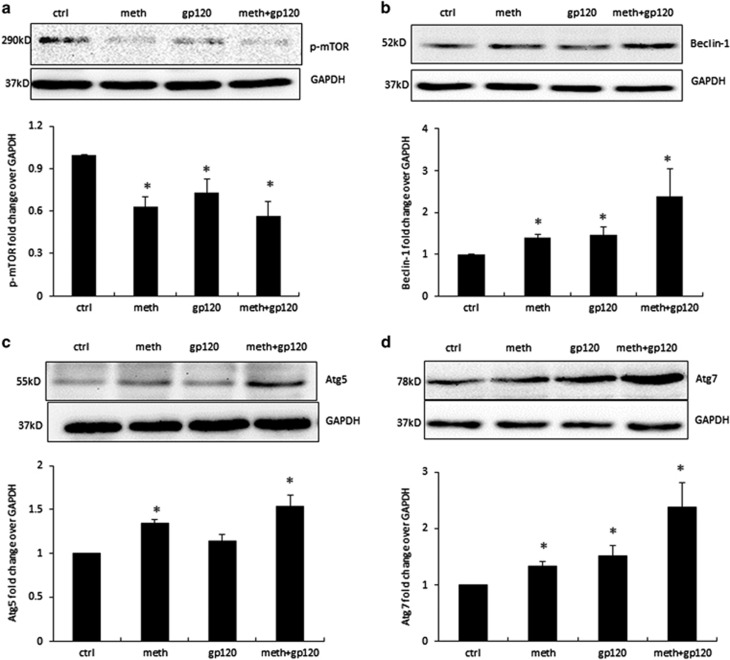
METH and gp120 IIIb induce autophagy through mTOR, Beclin-1 and Atg5/7 pathways. SVGA cells were treated with 1 mM METH and 400 pM gp120 IIIb for 24 h and were then subjected to western blotting to measure the signaling proteins p-mTOR (**a**), Beclin-1 (**b**), Atg5 (**c**) and Atg7 (**d**). The results are shown as mean±S.E. from three independent experiments. Data quantified by AlphaEase FC software are shown at the bottom of each panel. *P<0.05

To determine the signaling molecules involved in METH- and gp120-induced autophagy, we treated SVGA cells with 1 mM of METH, 400 pM of gp120 IIIb, and combination for 24 h. mTOR is one of the upstream protein that has key role in initiation of autophagy. Activation of mTOR by phosphorylation suppresses autophagy, whereas its dephosphorylation leads to initiation of autophagy. Our results showed significant downregulation of p-mTOR level, when the SVGA cells were treated with METH and gp120 IIIb (Figure 2a). Beclin-1 is one of the most important proteins that initiate autophagosome formation. Our results showed that METH and gp120 IIIb significantly upregulate Beclin-1 level, suggesting the role of Beclin-1 in METH- and gp120-mediated autophagy (Figure 2b). In addition, signaling molecules Atg5 and Atg7 have been reported to be involved in elongation of autophagosome. In METH- and gp120-treated cells, both Atg5 and Atg7 were induced, suggesting the involvement of Atg5 and Atg7 in METH- and gp120-induced autophagy in astrocytes (Figures 2c and d).

**Figure 3:**
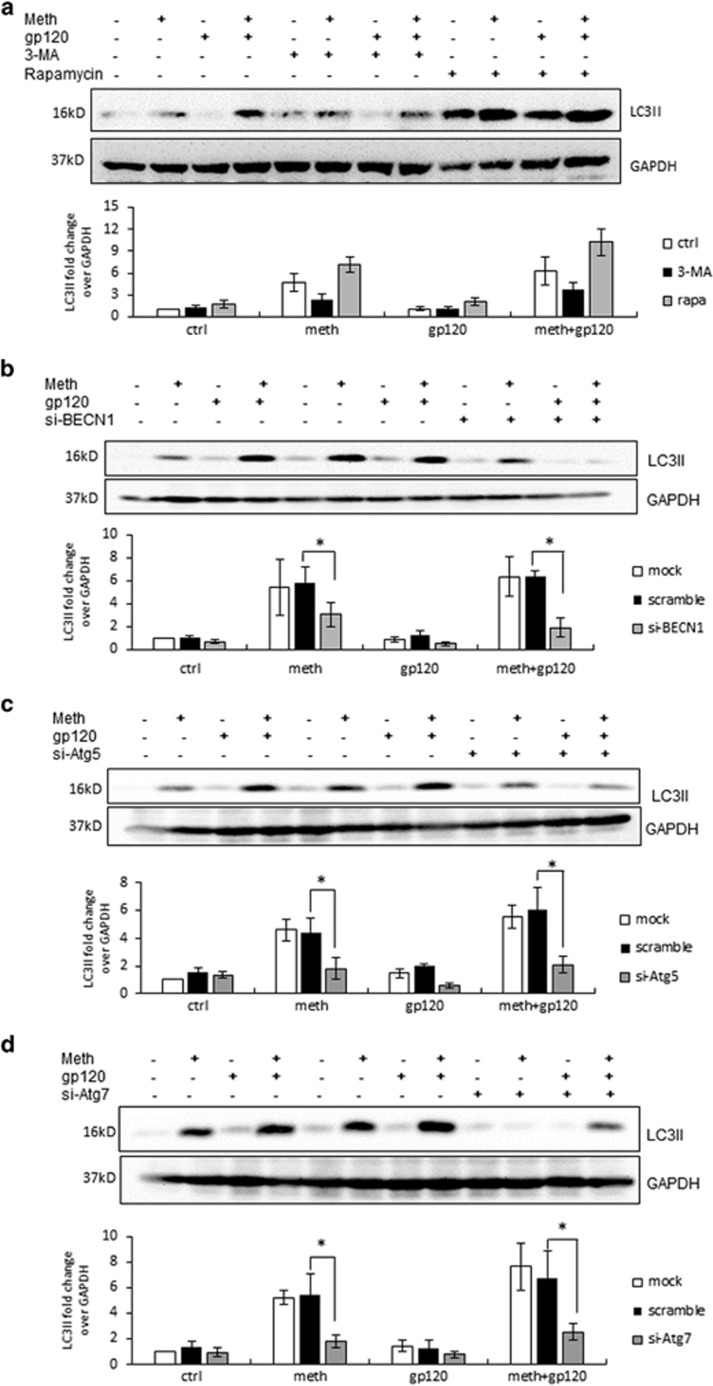
Involvement of signaling proteins mTOR, PI3K, Beclin-1, Atg5 and Atg7 as confirmed by the use of chemical inhibitors and specific siRNAs. The results are shown as mean±S.E. from three independent experiments. Data from the western blot of LC3II was quantified by AlphaEase FC software and are shown at the bottom of each panel. *P<0.05. (**a**) SVGA cells were treated with or without 3-MA or rapamycin, and then treated with 1 mM METH and 400pM gp120 IIIb for 24 h. (**b**) SGVA cells transfected with Beclin-1 siRNA or a scrambled siRNA were treated with 1 mM METH and 400pM gp120 IIIb for 24 h. (**c**) SGVA cells transfected with Atg5 siRNA or a scrambled siRNA were treated with 1 mM of METH and 400pM of gp120 IIIb for 24 h. LC3II was detected by western blot. (**d**) SGVA cells transfected with Atg7 siRNA or a scrambled siRNA were treated with 1 mM of METH and 400 pM of gp120 IIIb for 24 h

To further investigate METH- and gp120-induced autophagic pathway, we treated cells with 3-methyladenine (3-MA), a phosphoinositide 3-kinase (PI3K) inhibitor. PI3KIII complexes with Beclin-1 and functions as one of the key regulators of autophagy. As shown in Figures 3a, 3-MA decreased the expression of METH- and gp120-induced LC3II. As expected, pretreatment of the cells with mTOR inhibitor rapamycin caused >60% increase in the level of LC3II. Furthermore, to confirm the role of Beclin-1, Atg5 and Atg7 in METH- and gp120-induced autophagy, we used specific siRNAs of these proteins (Supplementary Figure 1, Figures 3b–d). Beclin-1 siRNA caused ~50% reduction in the level of LC3II in METH- or gp120 IIIb-treated cells, whereas it caused >70% reduction when the cells were treated with both METH and gp120IIIb together. Similarly, Atg5 and Atg7 siRNAs reduced METH- and gp120-induced LC3II expression by >60%, whereas scrambled siRNA did not cause any significant change.

### METH induces autophagy via opioid receptor and mGluR5 receptor-mediated pathway

**Figure 4:**
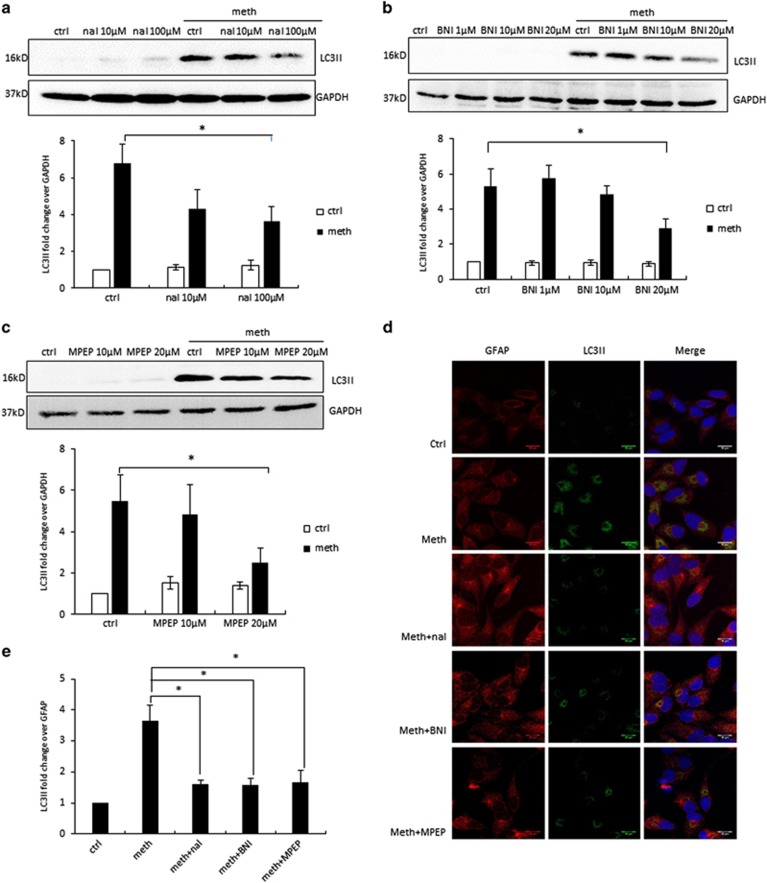

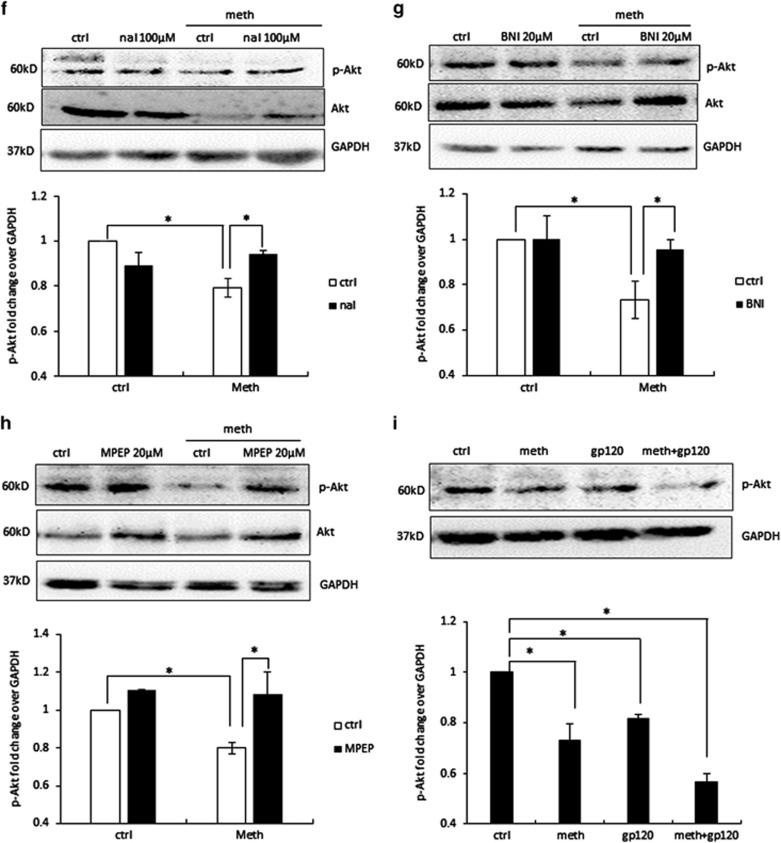
METH induces autophagy in astrocytes via opioid receptors and mGluR5 receptor. The results are shown as mean±S.E. from three independent experiments. Data from the western blot of LC3II (**a**–**c**) was quantified by AlphaEase FC software and are shown at the bottom of each panel. *P<0.05. For (**f**–**i**), cells were subject to western blot analysis with anti-Akt or anti-p-Akt antibody. (**a**) SVGA cells were treated with different concentrations of naltrexone, and then treated with 1 mM METH for 24 h. (**b**) SVGA cells were treated with different concentrations of nor-BNI, and then treated with 1 mM METH for 24 h. (**c**) SVGA cells were treated with different concentrations of MPEP, and then treated with 1 mM METH for 24 h. (**d**) SVGA cells were treated with or without naltrexone, nor-BNI, and MPEP, and then treated with 1 mM METH for 24 h. LC3II level was examined by confocal microscopy (scale bar, 20 *μ*m). (**e**) Confocal microscopy data quantified by ImageJ software. (**f**) SVGA cells were treated with or without 100 *μ*M naltrexone, and then treated with 1 mM METH for 24 h. (**g**) SVGA cells were treated with or without 20 *μ*M nor-BNI, and then treated with 1 mM METH for 24 h. (**h**) SVGA cells were treated with or without 20 *μ*M MPEP, and then treated with 1 mM METH for 24 h. (**i**) SVGA cells were treated with 1 mM METH and 400 pM gp120 IIIb for 24 h

Opioid receptor has been well documented in the literature for METH-mediated signaling that leads to neurotoxic effect.^35, 36, 37^ We therefore examined whether METH-induced autophagy was mediated by opioid receptor. Naltrexone is an antagonist that acts on multiple classes of opioid receptors, which shows most affinity to *μ*-opioid receptor, and to a much less extent, the *κ*-opioid receptor and *δ*-opioid receptor. To determine the effect of naltrexone on METH-induced autophagy, we treated SVGA cells with 10 or 100 *μ*M of naltrexone before METH treatment. Naltrexone blocked METH-induced LC3II expression with a significant decline in the LC3II level observed with 100 *μ*M of naltrexone treatment (Figure 4a). Another opioid receptor antagonist, nor-binaltorphimine dihydrochloride (nor-BNI), is highly selective for *κ*-opioid receptor. Pretreatment of nor-BNI reduced METH-induced LC3II expression in a concentration-dependent manner, with 20 *μ*M showing ~50% reduction in LC3II (Figure 4b). In addition to opioid receptors, metabotropic glutamate receptor type 5 (mGluR5) is considered to be associated with METH neurotoxicity. 2-Methyl-6-(phenylethynyl)pyridine (MPEP) at 20 *μ*M caused 55% reduction in METH-induced LC3II expression (Figure 4c). This result was further confirmed by immunocytochemistry in SVGA cells (Figures 4d and e). We also sought to determine whether combination of nor-BNI, naltrexone and MPEP would completely inhibit METH-induced autophagy. Our results showed that combination of the inhibitors did not cause any further reduction in LC3II level (Supplementary Figure 2). Therefore, our results suggest that METH may induce autophagy in astrocytes via alternative pathways.

After confirming the involvement of opioid and mGluR5 receptors, we next sought to investigate the involvement of the downstream signaling molecules of this pathway. The PI3K/Akt signaling pathway is upstream of mTOR, which is important for the regulation of autophagy. As shown in Figures 4f–h, treatment of METH caused downregulation of phosphorylated-Akt level. When cells were treated with opioid inhibitors or mGluR5 inhibitors before METH treatment, the decrease in p-Akt level was partially attenuated. Furthermore, we found that METH and gp120 IIIb together caused further decrease in p-Akt level (Figure 4i). Combined with previous finding that METH treatment showed significant decrease in p-mTOR level (Figure 2a), these results suggest that METH and gp120 IIIb together induce autophagy by inhibiting Akt/mTOR pathway.

### METH- and gp120-induced autophagy has pro-survival role against apoptotic cell death

**Figure 5:**
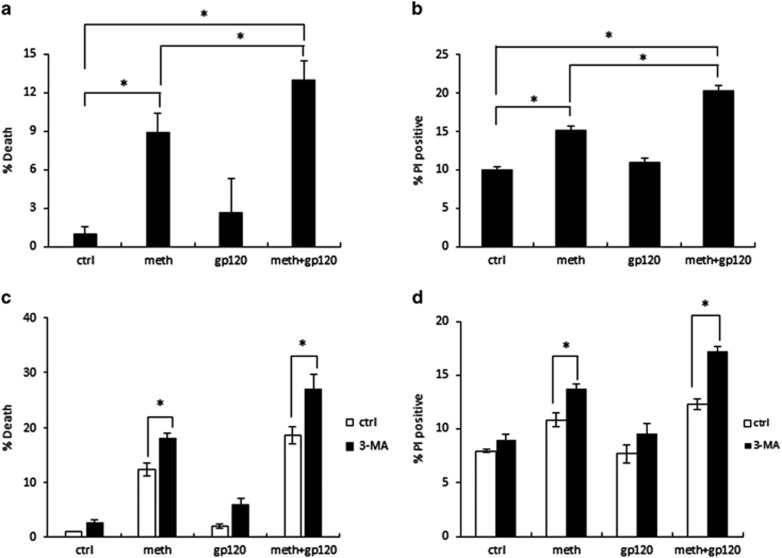
METH and gp120 IIIb induce cell death, which is exacerbated when autophagy is inhibited. Results are shown as mean±S.E. from three separate experiments. *P<0.05. (**a**) SVGA cells were treated with 500 *μ*M METH and 400 pM gp120 IIIb every 24 h for 72 h. The cells were subject to MTT assay. (**b**) SVGA cells were treated with 500 *μ*M METH and 400pM gp120 IIIb every 24 h for 72 h. The cells were subjected to PI staining. (**c**) SVGA cells were treated with 3-MA, and then treated with 500 *μ*M METH and 400 pM gp120 IIIb every 24 h for 72 h. The cells were subjected to MTT assay. (**d**) SVGA cells were treated with 3-MA, and then treated with 500 *μ*M METH and 400 pM gp120 IIIb every 24 h for 72 h. Then cells were subject to PI staining

Although autophagy is generally considered to be a protective mechanism, extensive autophagy can cause cell death, namely type II programmed cell death, or autophagic cell death. We therefore determined the role of autophagy in METH- and gp120-treated astrocytes. In preliminary experiments, we found that 24-h treatment of METH and gp120 is not sufficient to cause cell death. SVGA cells upon treatment of 500 *μ*M METH and 400 pM gp120 IIIb every 24 h caused cell death at 72 h as determined by MTT assay and propidium iodide (PI) staining (Figures 5a and b). Treatment of METH caused ~5% increase in cell death compared with control group, whereas treatment of gp120 alone did not cause any significant change in cell viability. However, when cells were treated with METH and gp120 simultaneously, a 10% increase in cell death was observed, which is statistically significant increase compared with either METH or gp120 group.

As cell death was confirmed in METH and gp120-treated cells, we next examined whether autophagy has pro-survival or pro-death role. Therefore, we treated cells with 3-MA, an autophagy inhibitor, before each dose of METH or gp120 IIIb for 72 h. The results showed that cell death induced by METH and gp120 was markedly increased by 3-MA treatment (Figure 5c and d). These findings suggest that inhibition of autophagy can exacerbate METH- and gp120-induced cell death, which further reveals that autophagy is a pro-survival mechanism with a long-term exposure to METH and gp120.

## Discussion

**Figure 6:**
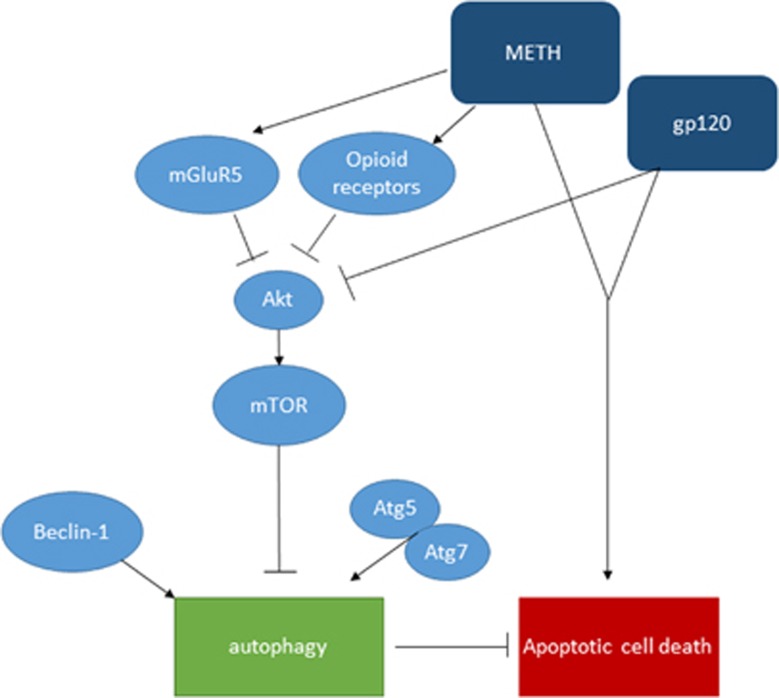
Schematic diagram showing the signaling pathways leading to METH- and gp120-induced autophagy. METH interacts with mGluR5 and opioid receptors and induce autophagy. METH and gp120 inhibit Akt, which in turn suppresses downstream signal mTOR, and activates Beclin-1- and Atg5/7-dependent autophagy pathway. METH and gp120 coordinate to induce cell death, which is exacerbated when autophagy is inhibited

The use of METH is highly prevalent among individuals infected with HIV-1, especially men who have sex with men.^38^ Previous studies have shown that use of METH exacerbates HIV-1 infection, accelerates the onset of HAND and causes resistance to ART treatment.^39^ Several recent studies have shown synergistic induction of proinflammatory cytokines and oxidative stress by METH and HIV-1 gp120 that leads to CNS injury.^23, 24, 40^ However, the role of autophagy in METH- and gp120-induced neurotoxicity remains poorly understood. This study reveals for the first time that METH and HIV-1 gp120 show additive effect in inducing autophagy in astrocytes. METH exerts its effect through *κ*- and *μ*-opioid receptors and mGluR5. METH and gp120 cause decrease in the level of downstream signaling protein Akt, thus inhibits mTOR and leads to the initiation of autophagy by regulating Beclin-1, Atg5 and Atg7 (Figure 6). This study also demonstrates that autophagy mitigates cell death induced by METH and gp120 in SVGA astrocytes. This is the first study that describes the complete mechanistic pathways involved in METH- and gp120-induced autophagy.

METH is self-administered, and typical doses for occasional users is 250–500 mg, and can reach as high as 1 g for chronic abusers.^41^ According to previous studies, 260 mg–1 g of METH in binge users will produce 17–80*μ*M blood METH concentrations.^42^ As METH is a small and lipid-soluble molecule, it is expected to distribute rapidly and extensively into high lipid-content tissues, such as brain. Therefore, the brain-to-serum concentration ratio in binge users can reach as high as 13 : 1, which means 200–1040 *μ*M of METH in the brain of binge users.^43^ In terms of gp120 IIIb, the dose used in the study (400 pM) matches well with the range of serum gp120 concentration measured in patients (100–800 pM).^44^ Therefore, the doses of both METH and gp120 IIIb used in this study are within physiological range and justifiable.

The development of addictive behavior of METH is regulated by mesolimbic dopaminergic system in the CNS.^45, 46^ In addition, other neurotransmitter systems including GABAergic, glutamatergic, and opioidergic systems, are also thought to be involved in the modulation of the addictive behavior.^47, 48, 49^ Previous studies have demonstrated that interactions between opioid receptors and dopamine receptors have a critical role in the addictive effect of drugs of abuse.^50^ Of different subtypes of opioid receptors, *μ*-opioid receptor has been shown to have modulatory role in METH-induced dopamine and serotonin metabolism in mice striatum.^51^ Pretreatment of naltrexone attenuated the expression of behavioral sensitization induced by METH in mice.^52^ Another opioid receptor, *κ*-opioid receptor is known to be involved in the modulation of addictive behavior following administration of psychostimulants.^53^ Apart from opioid receptors, mGluR5 has been found to be associated with neurocognitive impairments mediated by METH.^54, 55, 56, 57^ This study has demonstrated that both opioid receptors and mGluR5 interact with METH and initiate the downstream autophagy signaling cascade. As both opioid receptors and mGluRs are enriched in the CNS, it would be interesting to study the crosstalk between these receptors, and whether autophagy is associated with the behavioral modulating effects of drugs of abuse.

The kinase mTOR has a critical role in inducing autophagy. PI3K/Akt is a positive upstream regulator of mTOR, and activation of mTOR suppresses autophagy. In this study, we have shown that both METH and gp120 IIIb lead to a decrease in the level of total and phosphorylated-Akt, and that METH-induced reduction in p-Akt is abrogated by antagonists of both opioid receptors and mGluR5. Combining the fact that METH and gp120 cause downregulation of p-mTOR, it is reasonable to postulate that METH acts on both opioid receptor and mGluR5, and inhibits downstream signaling PI3K/Akt leading to deactivation of mTOR and initiation of autophagy. In addition, Beclin-1 and LC3 are the key proteins for normal function of autophagy. During autophagy, LC3I is converted to LC3II, which localizes on the surface of autophagosome. There is difference in the immunoreactivity of LC3I and LC3II, and the sensitivity of detection is much higher for LC3II than that for LC3I in most cases. Therefore, the comparison of LC3II level between different treatment groups is considered to be a more accurate method.^58^ Beclin-1 is essential for the formation of autophagosome, and has a crucial role in the maintenance of homeostasis and cellular housekeeping. The pro-autophagic activity of Beclin-1 can be attenuated by Bcl-2,^59^ and therefore, Beclin-1-Bcl-2 complex is thought to regulate the switch between autophagy and apoptosis.^60^ Our results showed that METH and gp120 cause increase the level of Beclin-1 in astrocytes, and knockdown of Beclin-1 significantly abrogated the change in LC3II level, suggesting the critical role of Beclin-1. As METH and gp120 contribute to astrocytic cell death, future study will focus on the interaction between Bcl-2 and Beclin-1 in determining the fate of cells under the stress of drugs of abuse.

There has long been a debate on the role of autophagy. It has been documented as a pro-survival mechanism for the cells that helps to maintain the nutrients level under starvation condition.^25^ However, there is conflicting evidence indicating that autophagy could be a pro-death mechanism.^61, 62, 63, 64^ Autophagic cell death was first defined morphologically for the cell death that shows appearance of numerous double-membrane vacuoles in the cytoplasm.^27^ However, one question that constantly arises is whether autophagy activity monitored in the dying cells is the cause of death or is the attempt to salvage the cells from other forms of cell death. In terms of drugs of abuse, it has been reported that suppression of autophagy precipitates neuronal cell death following low doses of METH treatment.^30^ Chronic treatment of morphine, has shown to induce cell death, which is further exacerbated by autophagy inhibition.^31^ In our study, there is ~5% cell death following 3-day METH treatment. Although gp120 alone does not cause any significant change in cell viability, it significantly promotes cell death when treated in combination with METH. In this case, the cell death is exacerbated when autophagy is blocked, suggesting that autophagy is an early response to the stress induced by METH and gp120, and has a protective role in this scenario.

Autophagy, besides a pro-survival mechanism against environmental stress, is also considered to be a defensive mechanism against invasion of microorganisms in innate and adaptive immunity.^25^ It has been reported that autophagosomes target and degrade a wide range of intracellular pathogens.^65, 66, 67^ Increasingly evident is that autophagy contributes to HIV-1 replication and progression.^68^ Some of the autophagy-associated genes have been correlated with HIV-1 replication as well. For example, shRNA knockdown of Atg4A, Atg5 and Atg16 has been shown to inhibit replication of HIV-1_LA1_ in SupT1 cells, without affecting cell viability.^69^ In another study, LC3 and Beclin-1 are found in complexes with HIV Gag and Nef, in which, Nef acts against the maturation of autophagosome and protects HIV from being degraded.^70^ In terms of HIV-associated neurologic diseases, increased autophagic markers were found in the brain tissues of patients with HIV-encephalitis and HAD.^71, 72^ As HIV-1 gp120 is found to induce autophagy, further study to visualize the localization of this viral protein would confirm the function of gp120 in autophagy initiation and maturation.

METH has been found to exacerbate HIV-induced cytotoxicity in many aspects.^24, 73^ METH and gp120 are known to cause oxidative stress, which takes place via altered expression of CYP450 and multidrug resistance protein-1.^24, 74^ In addition, oxidative stress has also been proved to have a role in the development of HAND.^75^ Previous reports have demonstrated that reactive oxygen species (ROS) generated in mitochondria represents one of the main inducers of autophagy.^76^ In response to ROS, preferential autophagy can occur, which clears damaged or excess organelles such as peroxisomes,^77^ endoplasmic reticulum (ER)^78^ and mitochondria.^79^ Other than that, oxidized proteins can be degraded by autophagy as well.^80^ In these cases, autophagy functions as a defense mechanism against oxidative stress. Therefore, it would be interesting to investigate the role of ROS in METH- and gp120-induced autophagy in astrocytes.

In summary, this study shows that METH and HIV-1 gp120 additively induce autophagy in astrocytes, and Akt, mTOR, Beclin-1, Atg5 and Atg7 are involved in the autophagic signaling pathway. Our study further suggests that combination treatment of METH and gp120 cause cell death, which is further exacerbated by autophagy inhibition. The study is clinically relevant because METH is highly prevalent in HIV-1 patients and METH is known to exacerbate HIV neuropathogenesis. Our study aids to the knowledge in HIV-1-associated neurotoxicity and a new direction for further study.

## Materials and Methods

### Cell culture and reagents

SVGA cells (astroglial cells modified from simian virus 40 (SV40)-transformed human glial cells (SVG)) were generously provided by Dr. Avindra Nath. Cells were grown in Dulbecco's modified Eagle's medium (DMEM) (Cellgro, Manassas, VA, USA) supplemented with 10% head-inactivated fetal bovine serum (FBS), 1% non-essential amino acids, 1% sodium bicarbonate, 1% l-glutamine and 50 *μ*g/ml of gentamicin. Human fetal astrocytes (HFA) were obtained from aborted fetal brain tissue from Birth Defect Research Laboratory (BDRL), Seattle, WA, USA. HFA were grown in DMEM media supplemented with 10% FBS and 1% gentamicin. The cells were maintained in an incubator at 37 ^°^C and humidified air with 5% CO_2_. The cells were seeded in six-well plates at a density of 0.8 × 10^6^ per well in 2 ml media and allowed to adhere overnight before treatment with METH and gp120 IIIb as specified in the figures. METH, PI, naltrexone and mGluR5 antagonist MPEP were obtained from Sigma-Aldrich (St. Louis, MO, USA). Recombinant HIV-1 gp120 IIIb (catalog number 11784) was obtained from the NIH AIDS Research and Reference Reagent Program. KOR antagonist nor-BNI was purchased from Tocris Bioscience (Ellisville, MO, USA). RIPA buffer was purchased from Boston BioProducts, Ashland, MA, USA (catalog number BP-115DG). Lipofectamine 2000 was purchased from Invitrogen Inc. (Carlsbad, CA, USA). The inhibitors against PI3K (3-MA) and mTOR (rapamycin) were obtained from Cayman Chemicals (Ann Arbor, MI, USA). Specific antibodies against LC3B, Atg5, Atg7, p-Akt, p-Bcl-2, p-mTOR and GAPDH were obtained from Cell Signaling Technology (Beverly, MA, USA). Vectashield Mounting Medium with DAPI was obtained from Vector laboratories (Burlingame, CA, USA). MTT assay kit was obtained from EMD Millipore (Billerica, MA, USA). Specific siRNAs of Beclin-1, Atg5 and Atg7, and control siRNA were purchased from Ambion Inc. (Carlsbad, CA, USA).

### Transfection of astrocytes with siRNA

Cells were plated at 0.6 × 10^6^ cells per well in a six-well plate. The cells were allowed to adhere overnight before transfection with 50 nM siRNA. Transfection of SVGA was performed using Lipofectamine 2000 as per the manufacturer's instructions. Briefly, complete medium was removed from plates, and cells were washed twice with phosphate-buffered saline (PBS) before addition of serum-free medium. Transfection reagent as mixtures of Opti-MEM, lipofectamine and siRNAs against Beclin-1, Atg5, Atg7 and control siRNA were prepared and added into the wells. After 24 h, the transfection reagent was replaced with fresh complete medium. Cells were trypsinized after 10 h and re-seeded into six-well plates at a density of 0.8 × 10^6^ per well. METH and gp120 IIIb treatments were performed the next day as described above. Transfection with 50 nM of scrambled siRNA was used as the negative control.

### Western blotting

SVGA cells were harvested at indicated time points in RIPA buffer (Boston BioProducts). The whole-cell lysates were homogenized and centrifuged for 15 min at 14 000 r.p.m. to obtain protein extracts. The protein concentration was measured using bicinchoninic acid (BCA) assay. In all, 40 *μ*g of protein was loaded in each well of 12% polyacrylamide gel for electrophoresis. The proteins were separated at 90 V for 2.5 h and transferred onto PVDF membrane at 350 mA for 90 min. The membranes were probed with appropriate primary and secondary antibodies for LC3II, Beclin-1, P-Bcl-2, p-mTOR, p-Akt, Atg5 and Atg7 to measure their expression levels. The bands were detected using BM Chemiluminescence Western Blotting Substrate (POD) (Roche Applied Sciences, Indianapolis, IN, USA). The bands were analyzed using AlphaEase FC software (Alpha Innotech, San Leandro, CA, USA), and the intensities of bands were normalized using GAPDH.

### Immunocytochemistry

SVGA cells were seeded at 0.8 × 10^6^ on 1.5 mm cover slips followed by treatment with METH and gp120 IIIb. After termination of the treatment, the cells were fixed with 1 : 1 ice-cold methanol and acetone solution for 20 min at –20 ^°^C. The wells were air dried followed by blocked and permeabilized with 1% BSA in PBS with 0.1% Triton for 30 min. After blocking, the cells were then incubated with a cocktail of rabbit anti-LC3B antibody (1 : 2000) and a mouse anti-GFAP antibody (1 : 1500) (Abcam, Cambridge, MA, USA) overnight in a humidified chamber. After three washes with 0.1% Triton in PBS, the cells were incubated in the dark chamber for 1 h with an anti-mouse antibody conjugated with Alexa Fluor 555 (1 : 2000) and an anti-rabbit antibody conjugated with Alexa Fluor 488 (1 : 2000) (Cell Signaling Technology) followed by three washes with 0.1% Triton in PBS. Finally, the cover slips were transferred onto glass slides with 10 *μ*l of Vectashield mounting reagent with DAPI. The microscopy analysis was performed using a Leica TCS SP5 II laser scanning confocal microscope. The images were captured using a 40X zoom lens and, ImageJ software was used to analyze the images and calculate the intensity. GFAP was used as housekeeping protein to normalize the intensity of the LC3II.

### Transmission electron microscopy

SVGA cells were treated with METH and gp120 IIIb as indicated, and fixed in 2.5% glutaraldehyde in 0.1M sodium cacodylade buffer at 4 ^°^C for overnight. Specimens were processed and observed under transmission electron microscopy in Electron Microscopy Laboratory in School of Dentistry, UMKC.

### Cell proliferation assay

Cells were plated in 12-well plates and treated with METH and gp120 IIIb for indicated time periods. Upon termination of the experiment, medium was removed, and cells were washed with PBS. Cells were incubated with 500 *μ*l of 0.2 mg/ml MTT solution at 37 ^°^C for 3 h. The supernatants were discarded and 500 *μ*l per well dimethyl sulfoxide was added. MTT assay was performed to measure cell viability using a microplate reader. Absorbance was obtained at 570 nm with a reference filter at 630 nm.

### Detection of cell death by PI staining

Cells were plated in 12-well plates and treated with METH and gp120 IIIb for indicated time periods. Upon termination of the experiment, cells were washed twice with PBS, digested with trypsin-EDTA solution and collected by centrifugation. Cells were further washed twice with ice-cold PBS, and incubated with 500 *μ*l of 0.8 *μ*g/ml PI. Flow cytometric analysis was performed to monitor the florescence of DNA-bound PI. All data were analyzed by BD FACSDiva software (BD Biosciences, San Jose, CA, USA).

### Statistical analysis

The statistical analysis was performed to represent the data in mean±S.E. values. Results were based on at least three independent experiments with individual experiments performed in triplicate. For the comparison between two groups, statistical analysis was performed using one-way ANOVA to calculate *P*-values, and *P*-value ⩽0.05 was considered statistically significant.

## Abbreviations

METH: methamphetamine
CNS: central nervous system
MAPLC3: microtubule-associated protein-1 light chain 3
HAD: HIV-associated dementia
HAND: HIV-associated neurocognitive disorders
mTOR: mammalian target of rapamycin
3-MA: 3-methyladenine
PI3K: phosphoinositide 3-kinase
nor-BNI: nor-binaltorphimine dihydrochloride
mGluR5: metabotropic glutamate receptor type 5
MPEP: 2-methyl-6-(phenylethynyl)pyridine
PI: propidium iodide
ART: antiretroviral therapy
ROS: reactive oxygen species
ER: endoplasmic reticulum
HFA: human fetal astrocytes
DMEM: Dulbecco's modified Eagle's medium
FBS: fetal bovine serum
BCA: bicinchoninic acid
PBS: phosphate-buffered saline
