# Supplementary figures and images for: Methamphetamine potentiates HIV-1 gp120-mediated autophagy via Beclin-1 and Atg5/7 as a pro-survival response in astrocytes
Source: Cell Death Dis. 2016 Oct 20;7(10):e2425–. doi: 10.1038/cddis.2016.317 (PMC5133984; doi:10.1038/cddis.2016.317)

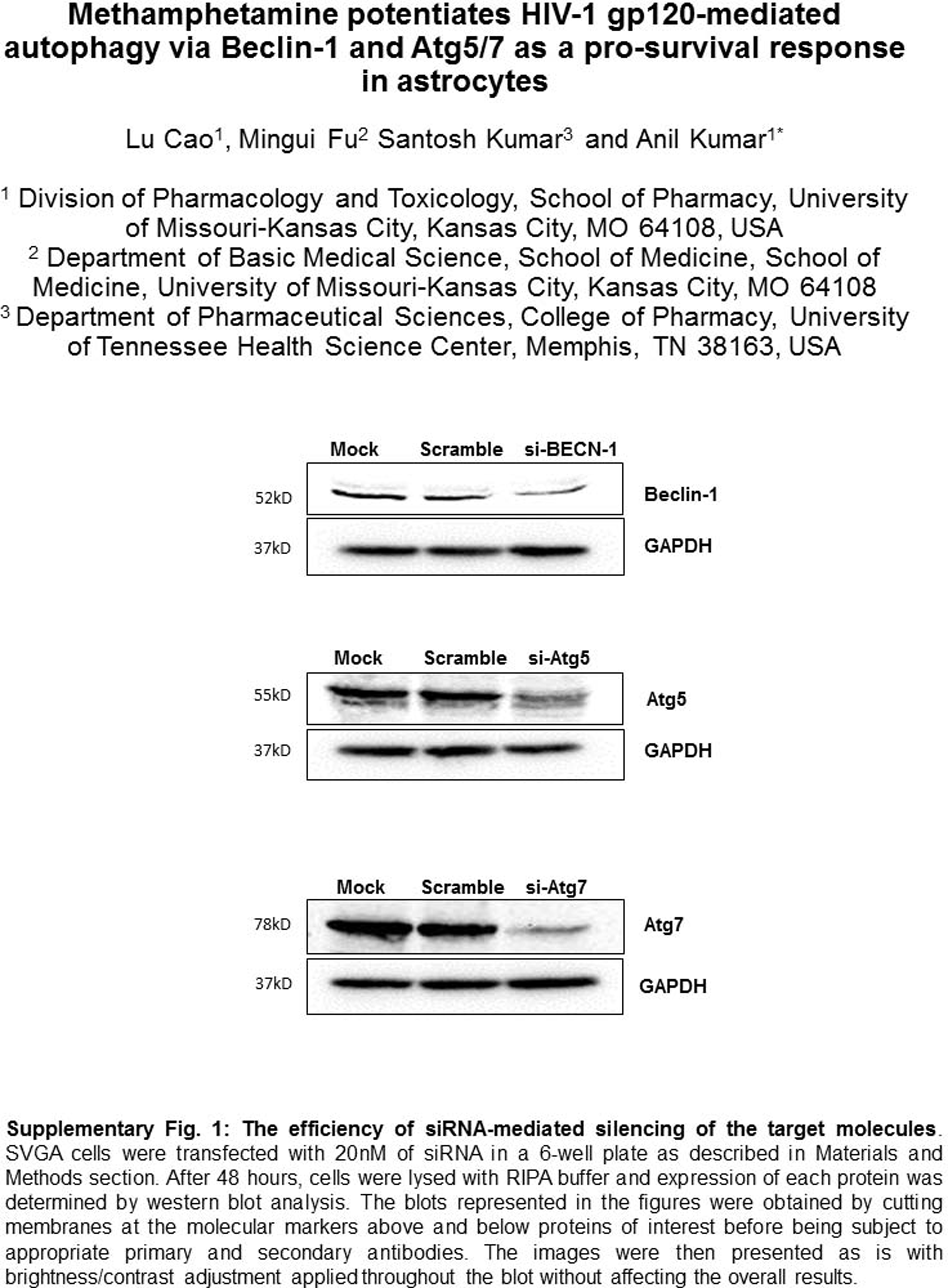

Supplement: Supplementary Figure 1 [file cddis2016317x1.tif]

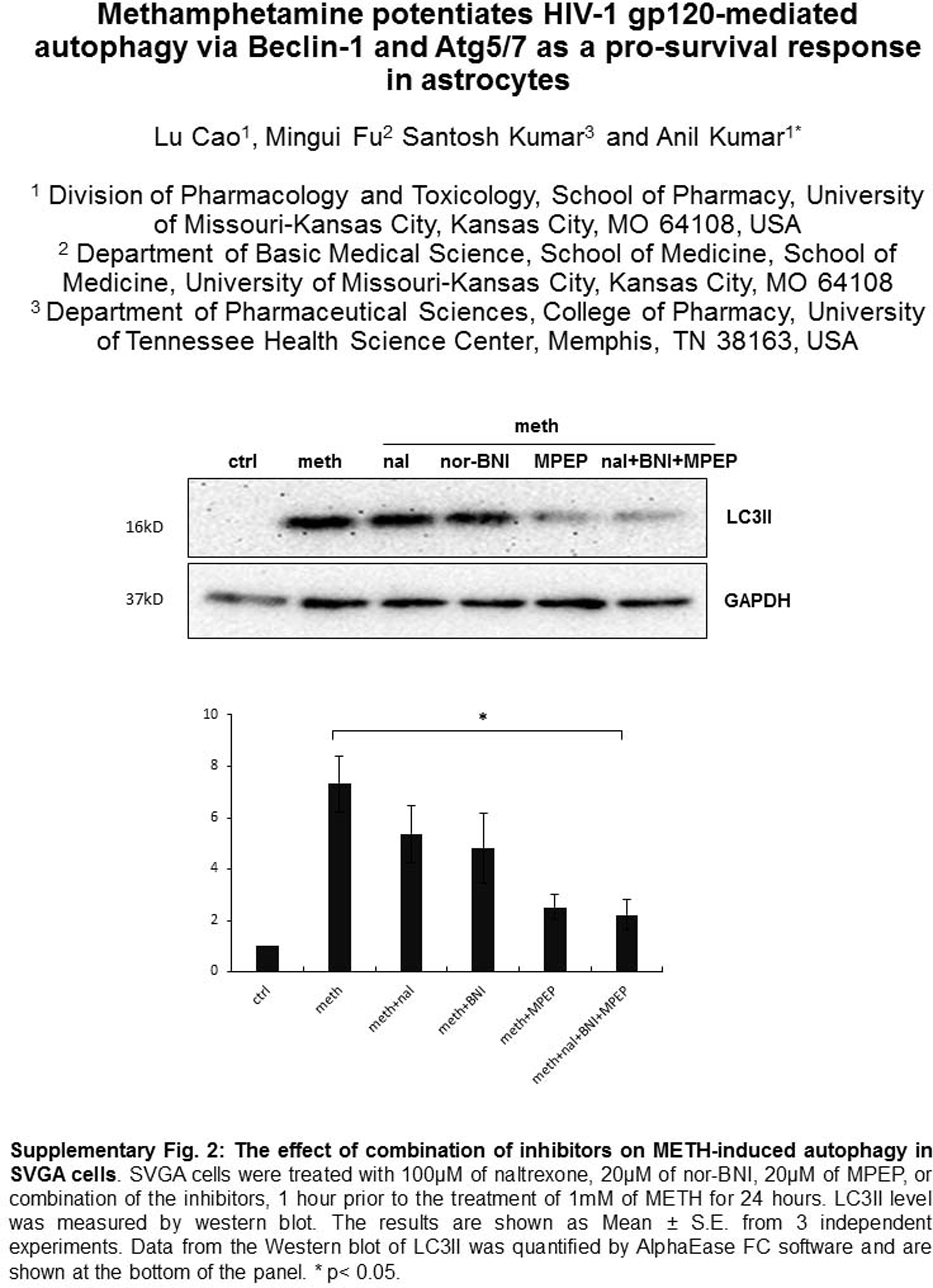

Supplement: Supplementary Figure 2 [file cddis2016317x2.tif]
